# Dual-Targeting CSC Therapy: Acid-Responsive Cisplatin/CaCO_3_@siRNA Nanoplatform Overcomes HCC Chemoresistance

**DOI:** 10.3390/ph19010022

**Published:** 2025-12-22

**Authors:** Fei Wang, Ming Lin, Yong Liu, Han Wang, Bin Li, Tan Yang, Weijie Li

**Affiliations:** 1 Department of Pharmacy, Tongji Hospital, Tongji Medical College, Huazhong University of Science and Technology, Wuhan 430030, China; 2 Department of Oncology, Renmin Hospital of Wuhan University, Wuhan 430060, China; feiwang2021@whu.edu.cn; 3 School of Pharmacy, Tongji Medical College, Huazhong University of Science and Technology, Wuhan 430030, China; liuyongswe@163.com (Y.L.); 15971506312@163.com (H.W.); libin@youdubio.com (B.L.); 4 Department of Pediatrics, Union Hospital, Tongji Medical College, Huazhong University of Science and Technology, Wuhan 430022, China; linming@hust.edu.cn

**Keywords:** drug delivery system, Bmi1, hepatocellular carcinoma, cancer stem cell, tumor microenvironment

## Abstract

**Background:** Cisplatin resistance is a major obstacle in the treatment of Hepatocellular carcinoma (HCC), characterized by reduced intracellular drug accumulation and altered DNA repair/apoptosis signaling. **Methods:** To address this challenge, we developed an acid-responsive nanoplatform consisting of a cisplatin-loaded CaCO_3_ core with a lipid coating that enables surface adsorption of Bmi1 siRNA, termed LCa/C@B. **Results:** These nanoparticles are subsequently coated with positively charged phospholipids, facilitating the absorption of Bmi1 siRNA. In vitro, LCa/C@B markedly enhanced intracellular cisplatin accumulation, downregulated Bmi1 and cancer stem cell (CSC) markers, and restored chemosensitivity in HepG2/MDR cells. In vivo, LCa/C@B achieved improved tumor localization, significant Bmi1 knockdown, suppression of CSC populations, and robust inhibition of tumor growth in a primary HCC model. Importantly, the dual-targeting design produced a synergistic therapeutic effect superior to free cisplatin or single-component formulations. **Conclusions:** This hybrid drug delivery system, combining calcium carbonate and cisplatin with Bmi1 siRNA, presents a promising approach for overcoming chemotherapy resistance in HCC.

## 1. Introduction

Hepatocellular carcinoma (HCC) represents one of the most prevalent primary liver cancers worldwide, known for its high fatality rate [1]. The pathogenesis of HCC involves a complex, multistage, and multifactorial process, characterized by multiple genetic or epigenetic alterations that result in the activation of oncogenes and/or the inactivation of tumor suppressor genes [2,3]. Common genetic abnormalities observed in HCC encompass mutations in p53 and beta-catenin, aberrant activation of receptor tyrosine kinases such as c-Met and IGF, abnormal methylation patterns of tumor suppressor genes APC and E-cadherin CpG islands, and dysregulated expression of oncogenes like Bmi1 [4,5]. These mutations or aberrant gene expression events frequently lead to abnormal hepatocyte proliferation or evasion of cell death, ultimately contributing to the development of HCC. The Bmi1 (B Mo-MLV insertion region 1) gene encodes a nucleoprotein with transcriptional inhibitory properties. This protein plays a crucial role in modulating cell proliferation, differentiation, and apoptosis by suppressing the expression of the Ink4A/Arf locus [6]. Overexpression of Bmi1 has been observed in various tumor types, including rectal cancer, medulloblastic carcinoma, gastric cancer, melanoma, breast cancer, prostate cancer, and non-small cell lung cancer [7,8,9,10]. Bmi1 acts not only as an oncogene, promoting tumorigenesis, but also as a key regulator of self-renewal in stem cells. Extensive research has demonstrated that Bmi1 is involved in the regulation of stem cells in various tissues, such as the nervous system, small intestine, and hematopoietic system [11,12,13]. Genetic knockout of Bmi1 has been shown to impede the proliferation of these stem cells, consequently affecting their self-renewal capabilities. Mechanistically, Bmi1 exerts its role in maintaining stem cell proliferation by inhibiting the Ink4A/Arf locus [14]. In the context of the liver, oval cells have been identified as putative hepatic stem cells or, at the very least, progenitor cells. Studies have shown that upregulation of Bmi1 expression in oval cells promotes their regenerative capacity, and this effect is also mediated through the inhibition of Ink4A/Arf expression [15]. Given its significant involvement in the initiation and progression of liver cancer, Bmi1 represents a promising target for gene therapy aimed at combating this devastating disease [16,17].

Cancer stem cells (CSCs) constitute a subset of tumor cells within tumor tissue that possess the remarkable ability to self-renew indefinitely and give rise to differentiated progeny [18,19]. CSCs exhibit enhanced clonogenicity and contribute significantly to tumor growth, metastasis, and recurrence. Therefore, targeting CSCs has emerged as a crucial therapeutic strategy in the management of tumors [20,21]. CSCs possess distinctive characteristics, including heightened expression of ABC transporters, acidic pH, infiltration of inflammatory mediators, and aberrant oxidative stress characterized by reactive oxygen species (ROS) [22,23,24,25]. The presence of CSCs in solid tumors often leads to the development of resistance following surgical resection and repeated radiotherapy, rendering tumor treatment ineffective. Consequently, overcoming CSC-mediated drug resistance represents a critical area of focus in tumor therapy [26,27]. The dysregulated response of CSCs to ROS represents a fundamental factor underlying drug resistance [28]. ROS response at lower levels promotes cell proliferation, whereas an elevated ROS response facilitates cell apoptosis [29]. Research indicates that heightened ROS response contributes to the aging of hematopoietic stem cells. In CSCs, increased ROS levels activate signaling pathways associated with aging and proliferation, subsequently impairing their self-renewal capacity [30]. Notably, the activation of p16^INK4a^, rb protein, and mitogen-activated protein kinase p38 exemplifies these mechanisms [31,32]. The response of tumor cells to ROS is augmented by both internal factors (such as inflammatory factors) and external factors (including radiotherapy and chemotherapy), inducing tumor cell apoptosis and thereby favorably influencing tumor treatment outcomes [33]. However, tumor cells, particularly CSCs, counteract ROS-induced cell death by upregulating antioxidant systems, thereby evading apoptosis and leading to the development of resistance against radiotherapy and chemotherapy [34,35,36]. Consequently, comprehending the mechanisms governing REDOX regulation in tumor stem cells represents a pivotal foundation for effectively addressing tumor resistance during treatment.

Due to impaired nutrient transport, rapidly proliferating tumor cells undergo a shift in their energy metabolism, commonly referred to as the Warburg effect [37,38]. This metabolic alteration leads to the accumulation of lactic acid as an extracellular metabolite, thereby lowering the pH of the tumor microenvironment (TME) [39]. Unlike normal tissues, which maintain a neutral pH of approximately 7.4, the extracellular space of tumor tissue is weakly acidic, with a pH range of 6.5 to 6.8 [40]. This acidic TME has provided opportunities for the development of acid-responsive intelligent nanodrug delivery systems [41,42,43]. However, since the reduction in lysosomal acidity is not exclusive to tumor cells, researchers primarily combine pH-responsive strategies with tumor cell-targeting strategies to achieve targeted molecular dissociation, controlled drug release, and improved drug distribution within tumor sites [44,45]. To exploit the weak acidity of the TME, acid-unstable chemical groups such as hydrazone, Schiff base, cis-maleate monoamide, and acetal are widely incorporated into the design of acid-responsive drug delivery systems [46,47,48,49]. Additionally, inorganic nanoparticles such as calcium carbonate nanoparticles (CaCO_3_ NPs) have the ability to degrade under acidic conditions, making them valuable components in the development of pH-responsive intelligent drug delivery systems [50,51,52].

**Scheme 1:**
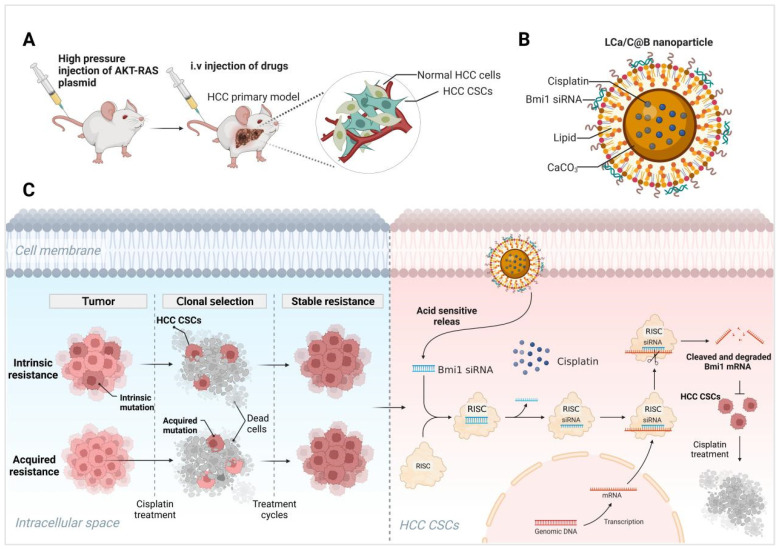
(**A**–**C**) LCa/C@B exhibits a synergistic effect in inhibiting CSCs, effectively reversing cisplatin chemotherapy resistance, and improving the therapeutic outcomes in HCC. Created in BioRender. Yang, T. (2025) https://BioRender.com/xp9brnm.

In this study, we synthesized and evaluated a novel drug delivery system in the shape of a watermelon, comprising a lipid-coated CaCO_3_/cisplatin hybrid nanoparticle combined with Bmi1 siRNA (LCa/C@B), with the aim of reversing cisplatin resistance by modulating CSC signaling in HCC (Scheme 1). The CaCO_3_/cisplatin hybrid nanoparticle (DCa/C) was synthesized using a reverse-phase microemulsion method, ensuring its uniform distribution within the CaCO_3_ nanoparticles. Positively charged phospholipids were then used to coat the DCa/C particles (LCa/C). Finally, Bmi1 siRNA was positively adsorbed onto the surface of the nanoparticles (LCa/C@B). This CaCO_3_/cisplatin hybrid nanoparticle exhibits several functions. Firstly, its core–shell structure enables efficient encapsulation of cisplatin, resulting in high drug loading efficiency. Secondly, when LCa/C@B encounters the acidic microenvironment of HCC, it undergoes disintegration, releasing CO_2_. This process breaks down the lipid layer, facilitating the release of small-sized cisplatin nanoparticles and Bmi1 siRNA into the deeper HCC tissue. Consequently, a synergistic effect is achieved, inhibiting cisplatin resistance. Lastly, the delivery of Bmi1 siRNA into deeper HCC tissues reduces cisplatin resistance by targeting CSCs, thereby enhancing the sensitivity of cisplatin chemotherapy. Overall, our results demonstrate that LCa/C@B degrades in the slightly acidic microenvironment, resulting in the simultaneous release of cisplatin and Bmi1 siRNA within cisplatin-resistant HCC cells. Moreover, LCa/C@B inhibits CSC growth and exhibits enhanced anti-tumor effects in cisplatin-resistant HCC cells. Furthermore, LCa/C@B demonstrates inhibitory effects in vivo HCC mouse model, along with the ability to suppress the expression of HCC CSCs.

## 2. Results and Discussion

### 2.1. Subsection Preparation and Characterization of LCa/C@B

**Figure 1:**
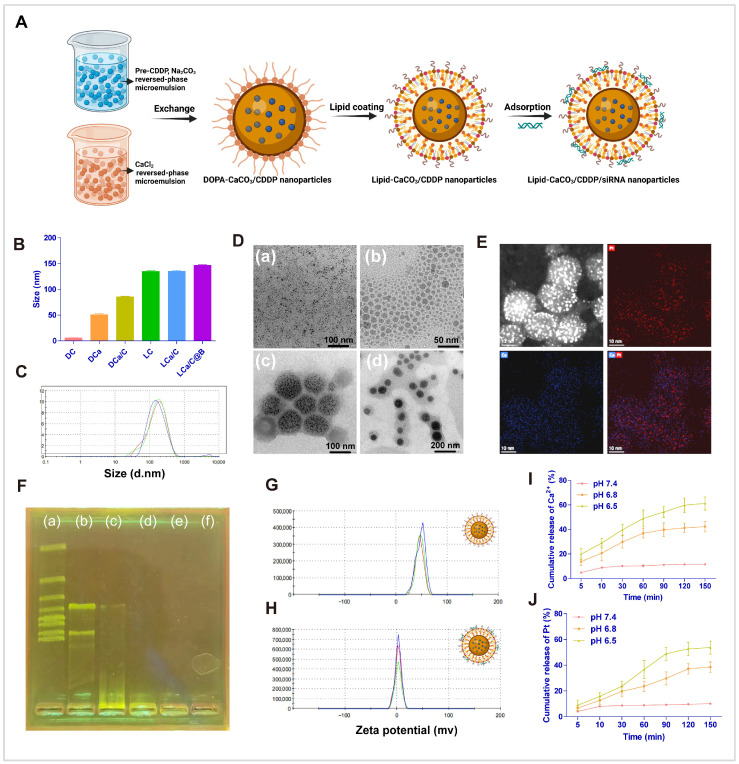
Characterization of nanoparticles. (**A**) Synthesis route of LCa/C@B. (**B**) Determination of nanoparticle size by DLS. DC (DOPA-cispaltin nanoparticle), DCa (DOPA-CaCO_3_ nanoparticle), DCa/C (DOPA-CaCO_3_/cispaltin nanoparticle), LC (lipid-cispaltin nanoparticle), LCa/C (lipid-CaCO_3_/cispaltin nanoparticle), and LCa/C@B (lipid-CaCO_3_/cispaltin@Bmi1 siRNA nanoparticle). (**C**) Determination of diameter distribution of LCa/C@B by DLS. (**D**) TEM images of nanoparticles. (a) DC, (b) DCa, (c) DCa/C, and (d) LCa/C. (**E**) EDX elemental detection of DCa/C. (**F**) (a–f) Agarose gel electrophoresis image illustrating Bmi1 siRNA loading efficiency at ratios from 60:1 to 300:1 (*w*/*w*). (**G**) Zeta potential of LCa/C detected by DLS. (**H**) Zeta potential of LCa/C@B detected by DLS. (**I**) Ca^2+^ released under different acidic conditions. (**J**) Pt released under different acidic conditions.

The reversed-phase microemulsion method has proven to be an excellent technique for preparing drug delivery systems. It enables the formation of nanoscale microreactors and precise control over nanoparticle morphology, resulting in drug delivery systems with controllable particle size, good dispersion, and lower impurity content [53,54]. In this study, the LCa/C@B drug delivery system was prepared using the reversed-phase microemulsion method (Figure 1A). The particle size and uniformity of the drug delivery system were analyzed using dynamic light scattering (DLS) detection. The average particle sizes of DC, DCa, DCa/C, LCa/C, LC, and LCa/C@B were measured to be 6 nm, 49 nm, 92 nm, 135 nm, 138 nm, and 151 nm, respectively (Figure 1B). The particle size distribution followed a normal distribution pattern (Figure 1C). The polydispersity index (PDI) values of LCa/C@B was 0.22 ± 0.03, indicating a relatively narrow size distribution (Table S1). Transmission electron microscopy (TEM) images revealed that DCa/C exhibited a heterozygous structure, with small-sized cisplatin nanoparticles evenly distributed within the CaCO_3_ nanoparticles, resembling watermelon seeds. The entire LCa/C exhibited a watermelon-like structure (Figure 1D). The successful encapsulation of cisplatin in the CaCO_3_ nanoparticles was confirmed by elemental mapping, which showed a uniform distribution of Pt and Ca elements inside the DCa/C (Figure 1E).

To determine the optimal preparation composition, LCa/C and siRNA were mixed at different ratios: 60:1, 120:1, 180:1, 240:1, and 300:1 (*w*/*w*). Agarose gel electrophoresis was performed to analyze the binding of siRNA to the nanoparticles. The results demonstrated that when the nanoparticles and siRNA were combined at a ratio of 180:1 (*w*/*w*), the siRNA was fully entrapped in the loading wells with LCa/C (Figure 1F). Following the loading of siRNA at a ratio of 180:1 (*w*/*w*), the zeta potential of LCa/C and LCa/C@B were measured to be 49.7 mV and 6.8 mV, respectively (Figure 1G,H). This difference can be attributed to the neutralization of the positive zeta potential from DOTAP by the siRNA, which carries a negative charge.

In order to investigate the acidity-responsive property of LCa/C nanoparticles, they were dispersed in buffers with different pH values. The release behavior of Ca^2+^ was evaluated, and the results showed that only 8.2% of Ca^2+^ was released after incubation in a pH 7.4 buffer for 150 min. In a pH 6.8 buffer, 10.9% of Ca^2+^ was released at 5 min and 39.8% was released at 150 min. The release rate and cumulative release amount were further improved in a pH 6.5 buffer, with 18.7% of Ca^2+^ released at 5 min and 58.9% released at 150 min (Figure 1I). These findings indicate that the release behavior of Ca^2+^ from LCa/C nanoparticles is dependent on acidity, with a more rapid and higher release rate achieved at lower pH values. To validate the disintegration of calcium carbonate in an acidic environment and the release of small cisplatin nanoparticles, the release of Pt was also tested. The results showed that 6.7% of Pt was released after incubation in a pH 7.4 buffer for 150 min. In a pH 6.8 buffer, 8.3% of Pt was released at 5 min and 38.7% was released at 150 min. In a pH 6.5 buffer, 9.2% of Pt was released at 5 min and 54.2% was released at 150 min (Figure 1J). The trend of Pt release was similar to that of Ca, indicating that cisplatin was released simultaneously with the disintegration of calcium carbonate under acidic conditions. These results confirm the successful synthesis of LCa/C@B nanoparticles, which exhibit remarkable uniformity and dispersion. Subsequently, the in vitro and in vivo biological functions of these nanoparticles were primarily evaluated.

### 2.2. The Uptake and Anti-Tumor Effect of LCa/C@B in Resistant HCC Cells

**Figure 2:**
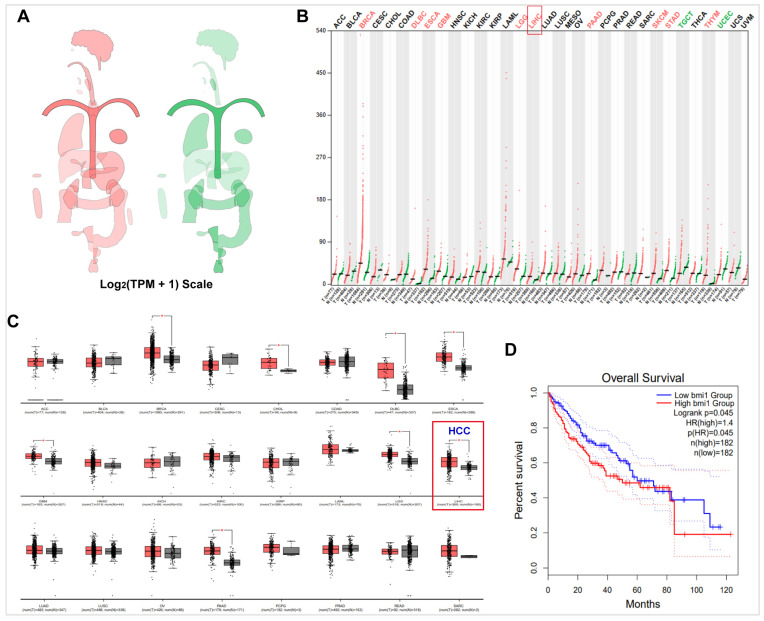
The expression of Bmi1 in HCC was detected by GEPIA. (**A**) The median expression of tumor and normal samples in body map. (**B**) The gene expression profile across all tumor samples and paired normal tissues (Dot plot). Each dot represents the expression of a sample. (**C**) The tissue-wise expression of Bmi1 in HCC cancer types using a box plot. (**D**) The survival analysis based on the expression status of Bmi1 and plot a Kaplan–Meier curve. *: *p* < 0.05.

HCC is a multifaceted disease characterized by numerous genetic and epigenetic changes. Among the genes implicated in this process is Bmi1, which encodes a transcriptional inhibitory nucleoprotein capable of suppressing the Ink4A/Arf gene locus, an essential tumor suppressor [55,56]. Through this mechanism, Bmi1 exerts control over cell proliferation, differentiation, and apoptosis [57]. Heightened expression of Bmi1 has been consistently observed across various tumor types. In order to assess the expression of Bmi1, we employed the GEPIA system. Analysis of the system’s Bodymap revealed a substantial upregulation of Bmi1 expression in cancerous tissues in comparison to normal tissues (Figure 2A). Specifically focusing on HCC tissues, we observed a significant increase in Bmi1 expression when compared to normal tissues (Figure 2B,C). Additionally, patients exhibiting elevated Bmi1 expression demonstrated markedly lower survival rates than those with lower Bmi1 expression (Figure 2D), thereby signifying an unfavorable prognosis for individuals with heightened Bmi1 expression. Bioinformatics analysis further bolstered the notion of elevated Bmi1 expression in HCC, with increased Bmi1 expression being significantly associated with diminished patient survival. Consequently, the development of therapies targeting Bmi1 holds paramount importance in the context of HCC treatment.

**Figure 3:**
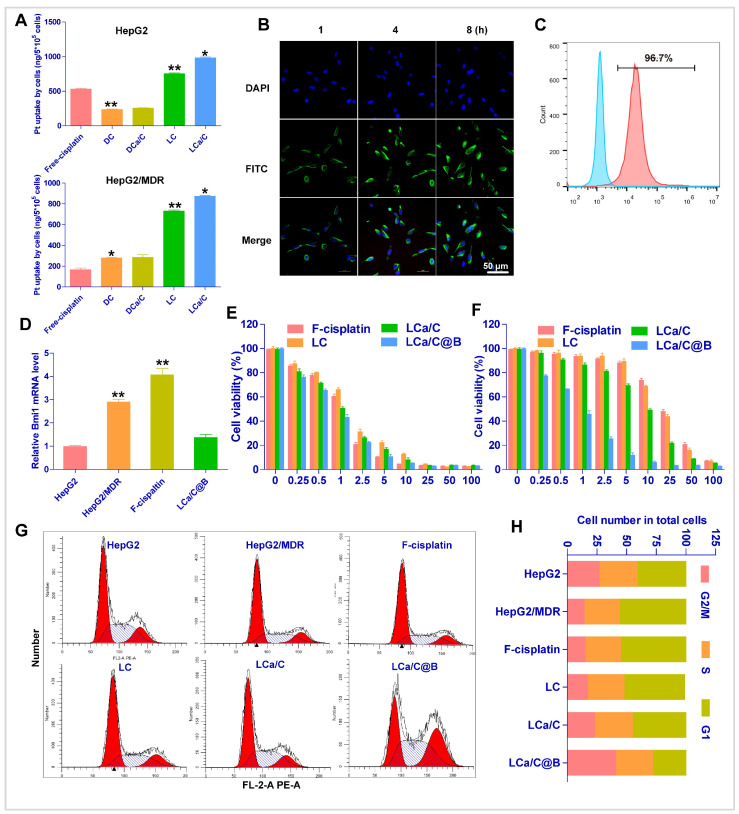
Cellular uptake and anti-tumor effects of particles in vitro. (**A**) Uptake of free cisplatin, DC, DCa/C, and LCa/C in HepG2 and HepG2/MDR cells at 48 h. (**B**) Fluorescence microscopy images showing the uptake of LCa/C@B at different time points (1 h, 4 h, and 8 h). (**C**) Tumor cell uptake was measured quantitatively by flow cytometry after 4 h treatment with LCa/C@B. (**D**) RT-PCR of Bmi1 mRNA expression in HepG2, HepG2/MDR, and LCa/C@B-treated HepG2/MDR cells. (**E**,**F**) Cell viability of HepG2 and HepG2/MDR cells treated with different doses of free cisplatin, LC, and LCa/C in HepG2 and HepG2/MDR cells for 24 h. (**G**,**H**) Cell cycle profile of HepG2/MDR cells treated with PBS, free cisplatin (5 μM), LC (5 μM cisplatin), LCa/C (5 μM cisplatin), and LCa/C@B (5 μM cisplatin and 50 nM Bmi1 siRNA) for 24 h. Cells were fixed with ethanol and labeled with propidium iodide, then analyzed for fluorescent DNA content with flow cytometry. Data are presented as mean ± SD of three independent experiments. *: *p* < 0.05; **: *p* < 0.01.

Chemotherapy resistance is a major cause of treatment failure following multiple rounds of treatment in HCC. One of the main mechanisms underlying chemotherapy resistance in HCC cells is the reduced uptake and concentration of chemotherapeutic agents in drug-resistant tumor cells [28,58]. To investigate the uptake of nanoparticles in drug-resistant HCC cells, we treated both normal HepG2 cells and cisplatin-resistant HepG2 cells (HepG2/MDR) with free cisplatin, DC, DCa/C, LC, and LCa/C, and measured the concentration of Pt using ICP-MS. Our results revealed that the concentration of Pt in HepG2 cells incubated with free cisplatin, DC, and DCa/C were similar at approximately 508 ng/5 × 10^5^ cells. In contrast, the concentration of Pt in HepG2/MDR cells was 97, 265, and 276 ng/5 × 10^5^ cells, respectively. However, cationic lipid encapsulation by LC and LCa/C greatly increased the uptake of cisplatin by drug-resistant cells, and the results showed that the uptake of cisplatin in HepG2/MDR cells could be increased to 809 ng/5 × 10^5^ cells (Figure 3A). These results indicate that lipid coating on the surface of nanoparticles, especially modification of positively charged lipid materials, can significantly increase the uptake of nanoparticles by tumor cells, enhance the uptake of chemotherapy drugs by drug-resistant tumor cells, and increase the accumulation of drugs in cells. The results of our study also demonstrate that HepG2/MDR cells exhibited reduced uptake of cisplatin. However, this effect was reversed upon treatment with LCa/C. Small interfering RNAs (siRNAs) have attracted considerable research attention for their potential therapeutic applications. Notably, several studies have explored the use of siRNAs to silence the expression of oncogenes and specific targets that promote the proliferation of tumor cells as an anticancer strategy. To assess the uptake efficiency of Bmi1 siRNA delivered by LCa/C@B, we conducted an experiment using FITC-labeled Bmi1 siRNA LCa/C@B. The results of fluorescence microscopy revealed a significant increase in the fluorescence intensity of LCa/C@B, beginning at the 4th hour of treatment, indicating its effective uptake by the cells (Figure 3B). Moreover, the uptake rate of tumor cells for LCa/C@B reached 96% as measured by flow cytometry after the 4th hour of treatment (Figure 3C). This high uptake efficiency, together with the subsequent functional knockdown of Bmi1, indicates that the delivered siRNA successfully reached the cytoplasm.

The treatment of HCC with cisplatin often results in two types of drug resistance: acquired and intrinsic [59]. However, increasing drug uptake can enhance the accumulation of cisplatin in drug-resistant tumor cells and improve the acquired drug resistance of HCC cells to some extent. Therefore, a therapeutic regimen that addresses intrinsic cisplatin resistance in HCC and comprehensively improves cisplatin resistance is necessary [60]. CSCs are important factors in the occurrence and development of HCC and are the main cause of chemotherapy resistance in HCC. Bmi1 is one of the main genes that upregulate the occurrence of HCC CSCs. Therefore, inhibiting Bmi1 gene expression using Bmi1 siRNA can inhibit HCC stem cells and reverse the intrinsic drug resistance of HCC. Consequently, we hypothesize that the combination of LCa/C and Bmi1 siRNA can effectively reverse both acquired and intrinsic cisplatin resistance in HCC. RT-PCR showed higher expression of Bmi1 in HepG2/MDR cells compared to HepG2 cells, and LCa/C@B significantly decreased the Bmi1 expression in HepG2/MDR cells (Figure 3D). These results suggest that LCa/C can effectively deliver Bmi1 siRNA to drug-resistant HCC cells and regulate the expression of Bmi1. Subsequently, we assessed the inhibitory effect of LCa/C@B on HepG2 and HepG2/MDR cells in vitro. The IC_50_ values of free cisplatin, LC, LCa/C, and LCa/C@B for HepG2 cells were 1.29, 1.53, 1.59, and 1.27 μg/mL cisplatin, respectively (Figure 3E). For HepG2/MDR cells, the IC_50_ values were 25.92, 15.64, 10.53, and 1.65 μg/mL cisplatin, respectively (Figure 3F). The cytotoxicity of free cisplatin, LC, LCa/C, and LCa/C@B demonstrated a dose-dependent effect. These results suggest that HepG2/MDR cells exhibit drug resistance to cisplatin treatment. LC and LCa/C improved cisplatin resistance to some extent and enhanced the killing effect of cisplatin on drug-resistant cells. However, when combined with Bmi1, LCa/C@B induced the highest amount of cell death compared to the free cisplatin, LC, and LCa/C treatment groups in HepG2/MDR cells. Consequently, LCa/C@B significantly enhanced cisplatin’s ability to kill cisplatin-resistant cells.

To further investigate the effect of LCa/C@B on drug-resistant cell line proliferation, we examined the effects of LCa/C@B on the cell cycle progression of HepG2/MDR cells. Cell cycle analysis by FACS showed that compared with HepG2 cells, the number of cells in the G1 phase of the cell cycle increased in drug-resistant HepG2/MDR cell lines, and HepG2/MDR cells treated with LCa/C@B were arrested in the G2 phase (Figure 3G). Quantitative results showed a significant increase in the number of HepG2/MDR cells in the G2 phase after LCa/C@B treatment compared with other groups (Figure 3H). The results also indicated that LCa/C combined with Bmi1 siRNA could significantly affect the cell cycle of drug-resistant HCC cells and cause cell cycle arrest.

### 2.3. LCa/C@B Reversed Cisplatin-Resistant HCC Cells by Inhibiting CSC Expression

**Figure 4:**
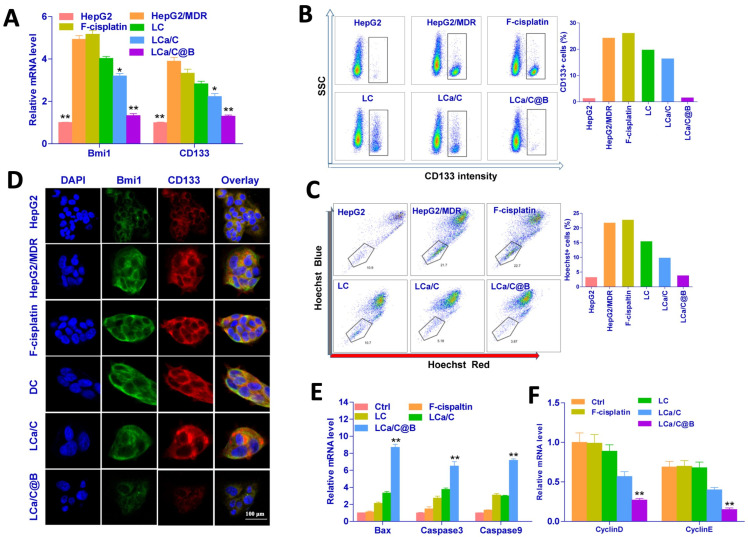
Mechanisms underlying the reversal of cisplatin resistance by LCa/C@B through CSC regulation in HepG2/MDR cells. (**A**) RT-PCR of Bmi1 mRNA expression in HepG2, HepG2/MDR, and free cisplatin, LC, LCa/C, and LCa/C@B-treated HepG2/MDR cells. (**B**,**C**) FACS analysis of CD133 and Hoechst staining in HepG2, HepG2/MDR, and treated with free cisplatin, LC, LCa/C, and LCa/C@B-treated HepG2/MDR cells for 24 h. (**D**) Immunofluorescence detection of Bmi1 and CD133 gene expression in HepG2, HepG2/MDR, and treated with free cisplatin, LC, LCa/C, and LCa/C@B-treated HepG2/MDR cells for 24 h. (**E**) RT-PCR detection of Bax, Caspase3 and Caspase9 in HepG2/MDR cells treated with different formulations for 24 h. (**F**) RT-PCR detection of Cyclin D and Cyclin E in HepG2/MDR cells treated with different formulations for 24 h. Data are presented as mean ± SD of three independent experiments. *: *p* < 0.05; **: *p* < 0.01.

Bmi1, as an important gene in the occurrence and development of HCC, can also affect the generation of chemotherapy resistance and treatment prognosis of HCC by regulating HCC stem cells. Therefore, we expect to reverse tumor resistance by inhibiting tumor stem cells with Bmi1 siRNA combined with LCa/C. CD133 is a recognized marker of HCC stem cells [61]. Hence, we demonstrated the mechanism related to the reversal of drug resistance by LCa/C@B through the inhibition of CD133 expression in drug-resistant HCC cells after Bmi1 inhibition. To investigate the mechanism underlying the reversal of tumor resistance by LCa/C@B, we treated HepG2/MDR cells with saline (control), free cisplatin, LC, LCa/C, and LCa/C@B (5 μM cisplatin and 50 nM Bmi1 siRNA). Firstly, we evaluated the expression of Bmi1 and CD133, the CSC markers, through RT-PCR analysis and Western blot. The results indicated that LCa/C@B significantly reduced the expression of Bmi1 and CD133 in HepG2/MDR cells compared to the control group (Figure 4A and Figure S1). Additionally, we performed flow cytometry to determine the effect of LCa/C@B on CD133-labeled cancer stem cells. The results revealed that the proportion of CD133-positive cells in HepG2/MDR cells greatly increased from 0.85% to 23.48% compared to HepG2 cells. This result indicates a significant increase in the proportion of CSCs in resistant HCC cells. Compared with the other intervention groups, the LCa/C@B group was able to reduce the proportion of CD133-positive cells to 1.34% (Figure 4B).

Similar results were obtained by Hoechst FACS analysis, which showed that LCa/C@B treatment suppressed the population of CSCs. Compared to HepG2/MDR and other treatment groups, the population of CSCs significantly decreased to 3.87% after treatment with LCa/C@B (Figure 4C). Furthermore, we used immunofluorescence to verify the expression of Bmi1 and CD133 in drug-resistant HCC cell lines after LCa/C@B intervention, and the results once again confirmed that the expressions of Bmi1 and CD133 were significantly reduced following LCa/C@B treatment (Figure 4D). Overall, these findings suggest that LCa/C@B can inhibit Bmi1 and reduce the level of HCC CSCs through Bmi1 inhibition in HCC cisplatin-resistant cell lines.

However, it remains unclear whether this regulation enhances the apoptosis-promoting and cell cycle-inhibition abilities of cisplatin. To investigate this, we first examined the expression of genes involved in apoptosis. Treatment with LCa/C@B significantly upregulated the expression of Bax, Caspase 9, and Caspase 3 in HepG2/MDR cells. In contrast, the expression levels of these genes were moderately increased in cells treated with LC and LCa/C. No significant changes in gene expression were observed in the other treatment groups (Figure 4E), indicating that LCa/C@B activated apoptosis in HCC-resistant cells. Consistently, RT-PCR results revealed that G1 phase-related cyclin D1 and cyclin E1 were downregulated by LCa/C@B treatment (Figure 4F). These findings collectively indicate that Bmi1 inhibition improved the anti-tumor effects and cell cycle arrest. In conclusion, these results suggest that the combination of Bmi1 siRNA and LCa/C can effectively regulate CSCs in HCC cisplatin-resistant cell lines. This combination approach promotes cisplatin-induced apoptosis of drug-resistant cells, representing the primary mechanism responsible for the reversal of cisplatin-resistant cell resistance.

### 2.4. LCa/C@B Suppress Tumor Growth in Genedrives’ Primary HCC Model in Mice

**Figure 5:**
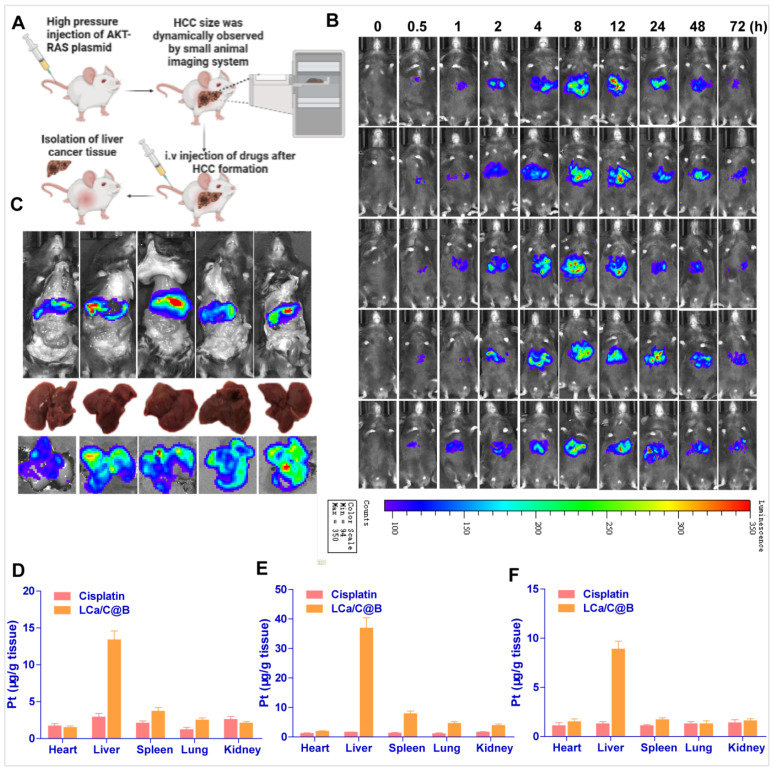
Distributions of Bmi1 siRNA and cisplatin delivered by LCa/C@B in primary HCC model mice. (**A**) Experimental procedure schematic. (**B**) Distribution of LCa/C@B in HCC tissue detected by a small animal imaging system. Bmi1 siRNA was labeled with Cy7.5. (**C**) Ex vivo bioluminescence imaging of tissues excised from the mice at 8 h after tail vein injection of LCa/C@B. (**D**–**F**) Pt distributions in mouse tissues at 3, 8, and 48 h after intravenous injection of cisplatin or LCa/C@B. Data are expressed as mean ± SEM (*n* = 5).

Tumor transplantation models have become a widely used tool for in vivo anti-tumor studies. However, it is important to consider the distinctions between subcutaneous and primary tumors. Firstly, subcutaneous tumors develop in an environment lacking supportive factors such as sufficient oxygen supply, angiogenesis, and nutrients for mesenchymal or fibroblastic cells. Secondly, the use of immune-deficient mice in subcutaneous tumor models fails to accurately simulate the body’s typical immune environment. Lastly, xenograft tumors solely consist of tumor cells and lack matrix components that may impact drug metabolism. In our investigation, we implemented an Akt/Ras plasmid-based in situ HCC model, which was constructed via high-pressure injection. The Akt gene induced adipogenesis, while the Ras gene induced liver cell mitosis. These two genes synergistically contributed to the development of HCC after a 6-week period (Figure 5A).

To determine whether LCa/C@B can enter tumor tissues, we examined the distribution of LCa/C@B in primary HCC model mice following drug administration. Using an in vivo imaging system, we found that LCa/C@B started to enter tumors (indicated by the red circle) at 0.5 h post-injection, and the amount of Bmi1 siRNA reached its peak at around 8 h after injection (Figure 5B). Additionally, LCa/C@B particles showed apparent accumulation in the liver, which potentially favors its efficacy in HCC treatment. Ex vivo observation of isolated organs at 8 h post-injection was consistent with the results of in vivo imaging, showing a high distribution of Bmi1 siRNA in liver tissues (Figure 5C). We then detected the distribution of cisplatin following LCa/C@B injection. At 3 h, 8 h, and 48 h after injection, the amount of Pt delivered by LCa/C@B was significantly higher than that delivered by free cisplatin in liver tissues, suggesting an excellent liver targeting effect of LCa/C@B (Figure 5D–F). At 48 h post-injection, cisplatin delivered by LCa/C@B still maintained a high concentration in the liver, indicating a long circulating property of LCa/C@B (Figure 5F). These results collectively demonstrate that LCa/C@B can efficiently deliver both cisplatin and Bmi1 siRNA into HCC tissues.

**Figure 6:**
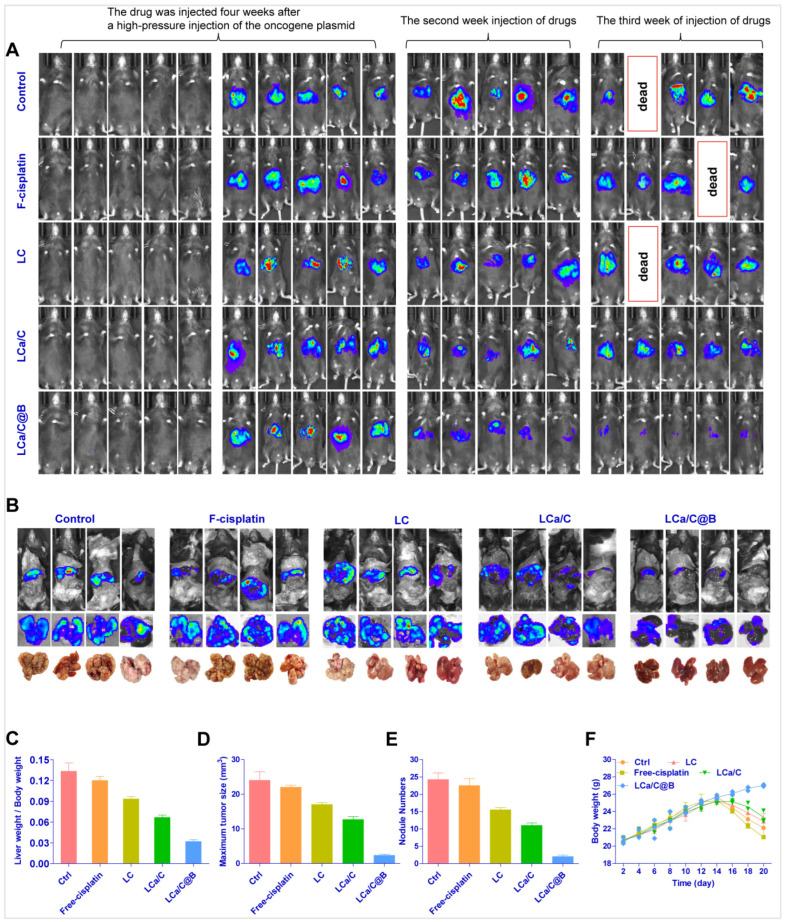
Anti-tumor effects of LCa/C@B in the primary HCC mouse model (equivalent to 1 mg/kg cisplatin and 170 μg/kg Bmi1 siRNA). (**A**) HCC tumor size detection in vivo detected by a small animal imaging system. Biotin was administered to HCC labeled with luciferase (a fragment expressing luciferase was inserted into the plasmid). (**B**) Excised livers at the endpoint of the experiment (the third week after the first injection of drugs). (**C**) Liver weight to body weight ratio in mice treated with different groups. (**D**) Maximum size of tumor nodules in mice liver after treatment. (**E**) Number of tumor nodules in the liver after treatment with different groups. (**F**) Body weight change curves of mice treated with different groups. Data are presented as mean ± SD (*n* = 4).

Following the formation of tumors, saline, free cisplatin, LC, LCa/C, and LCa/C@B were administered via tail vein injection four times per week over a one-week treatment period. The growth of HCC was monitored at regular intervals using a small animal imaging system. Multiple screenings and detection of tumor formation time showed that in situ HCC could form stably 4 weeks after the high-pressure injection of the oncogenic plasmid. Therefore, drug administration started 4 weeks after the plasmid injection (Figure 6A). In the LCa/C@B group, the fluorescence indicating HCC growth almost disappeared three weeks after injection, indicating that LCa/C@B had the strongest inhibitory effect on HCC growth. Mice in the saline, free cisplatin, and LC groups died, indicating poor prognosis after treatment with free cisplatin and LC (Figure 6A). To further verify the influence of drug therapy on HCC, mouse livers were dissected after administration for comparison, and various indexes of liver and HCC tissues were measured. The phenotype of mouse livers showed that the liver surface in the LCa/C@B treatment group was smooth, indicating a better prognosis (Figure 6B). The liver weight ratio, which represents the prognosis after drug chemotherapy, was the smallest in the LCa/C@B group (0.13) compared to the control (0.03), free cisplatin (0.11), LC (0.09), and LCa/C (0.06) treatment groups (Figure 6C). The maximum tumor size in the LCa/C@B group was significantly smaller than that of the other groups (Figure 6D). The number of tumor nodules in the LCa/C@B group (2 nodules) was significantly lower than that in the control (22 nodules), free cisplatin (21 nodules), LC (15 nodules), and LCa/C (10 nodules) groups (Figure 6E). Collectively, these data suggest that LCa/C@B significantly inhibited HCC growth and generated an enhanced anti-tumor effect in this mouse model. The weight of mice in the control, free cisplatin, LC, and LCa/C groups declined significantly after 12 days of treatment. In contrast, the weight of mice in the LCa/C@B group remained relatively stable throughout the treatment period (Figure 6F). This observation suggests that LCa/C@B could improve the treatment prognosis of primary HCC. Due to the rapid growth of HCC, which causes abnormal metabolism of liver function, the mice exhibited obvious weight loss.

**Figure 7:**
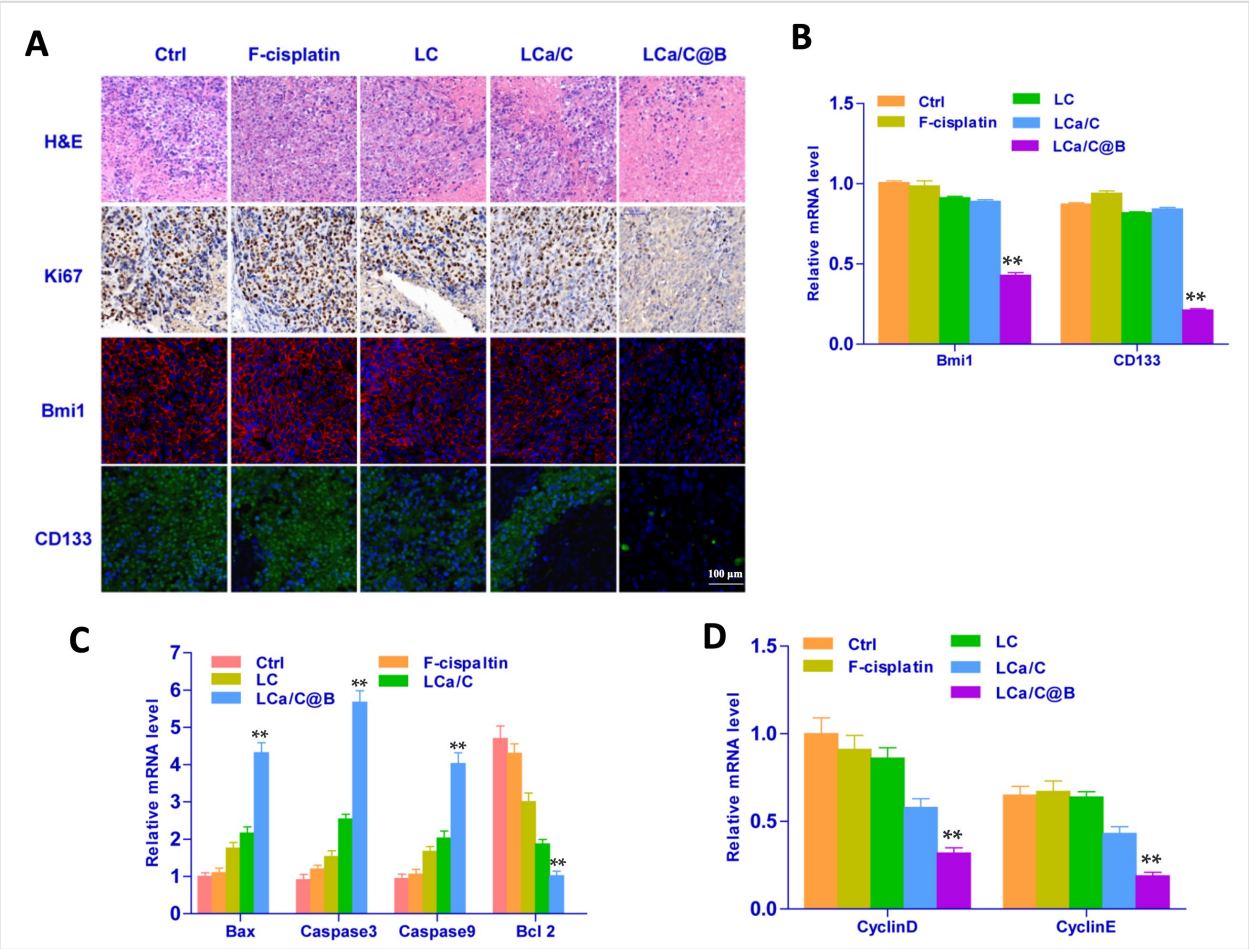
(**A**) Fluorescent images of immunohistochemical and histological examinations with Ki67, Bmi1, and CD133 in the liver (Ki67 in brown, Bmi1 in red, CD133 in green, and nuclei in blue). (**B**) RT-PCR detection of Bmi1, and CD133 in tumor tissues treated with different groups. (**C**) RT-PCR detection of apoptosis signal in tumor tissues treated with different groups. (**D**) RT-PCR detection of cell cycle signal in tumor tissues treated with different groups. The data are expressed as mean ± SD (*n* = 4). **: *p* < 0.01.

After conducting the animal pharmacodynamics experiment, we performed immunofluorescence, H&E staining, and gene expression analysis on HCC tissues. The H&E staining results indicated that the liver tissue remained in good condition following treatment with LCa/C@B in the primary HCC mice model. Furthermore, the livers of mice in the primary HCC model showed a significant improvement (Figure 7A). These findings suggest that LCa/C@B can improve the prognosis of this in situ HCC model. Immunofluorescence analysis revealed a significant reduction in the signaling of Ki67, Bmi1, and CD133 caused by LCa/C@B (Figure 7A). In addition, RT-PCR were performed to detect the gene expression of Bmi1 and CD133. The results indicated that LCa/C@B noticeably reduced their expression compared to the other treatment group (Figure 7B). These findings suggest that LCa/C@B can inhibit tumor proliferation and the expression of HCC CSCs in vivo. Subsequently, we examined the expression of genes involved in apoptosis and the cell cycle. The results showed that treatment with LCa/C@B significantly upregulated the expression of Bax, Caspase 9, and Caspase 3, while downregulating the expression of Bcl-2 in primary mice HCC tissue. However, in cells treated with LCa/C, the expression levels of Bax, Caspase 9, and Caspase 3 moderately increased. No significant changes in gene expression were observed in the other treatment groups (Figure 7C), indicating that LCa/C@B activated more apoptosis in vivo. Finally, the expression of cell cycle genes revealed that cyclin D1 and cyclin E1, which are related to the G1 phase, were downregulated by LCa/C@B treatment (Figure 7D). Taken together, these results indicate that Bmi1 inhibition improved the anti-tumor effects and caused cell cycle arrest in vivo.

## 3. Materials and Methods

### 3.1. Chemicals and Reagents

All solvents and reagents were used as received without further purification. Cisplatin was obtained from Sino-Platinum Metals (Guiyang, Guizhou, China). mPEG2000-silane, DOTAP, TritonX-100, hexanol, and cyclohexane were purchased from Sigma-Aldrich (St. Louis, MO, USA). The Bmi1 siRNA sequences for mice were as follows: sense strand 5′-CCA GAC CAC UCC UGA ACA UTT-3′ and anti-sense strand 5′-AUG UUC AGG AGU GGU CUG GTT-3′. For human, the sequences were: sense strand 5′-CCA GAC CAC UAC UGA AUA UAA-3′ and anti-sense strand 5′-UUA UAU UCA GUA GUG GUC UGG UU-3′.

### 3.2. Cell Culture and Cisplatin-Resistant Cells

The human tumor cell line HepG2 was obtained from the China Center for Type Culture Collection at Wuhan University (Wuhan, China). HepG2 and HepG2/MDR cells were cultured in high-glucose Dulbecco’s modified Eagle’s medium (DMEM) supplemented with 1% streptomycin, 1% penicillin, and 10% fetal bovine serum (FBS) in a 5% CO_2_ incubator at 37 °C. MDR/HepG2 cells were generated by treating HepG2 cells with 7.5 μg/mL (25 μM) of free cisplatin for 24 h, followed by culture in medium containing 3 μg/mL cisplatin (10 μM) for 10 days [62]. Subsequently, the cells were treated with 10 μg/mL free cisplatin for an additional 24 h. Surviving cells were cultured for 10–14 days with 7.5 μg/mL of cisplatin until the cells maintained steady growth for five consecutive passages. It is important to note that HepG2/MDR cells were washed for at least two passages before experiments to reduce cisplatin accumulation in cells.

### 3.3. Synthesis the Cisplatin Precursor of Cis-[Pt(NH_3_)_2_(H_2_O)_2_](NO_3_)_2_

The synthesis of the cisplatin precursor, cis-[Pt(NH_3_)_2_(H_2_O)_2_](NO_3_)_2_, was conducted following a procedure described in the literature. Initially, a suspension of 0.20 mmol cisplatin (1 mL) was prepared, and then 0.39 mmol of AgNO_3_ was added to the suspension under dark conditions. The mixture was subsequently protected from light and heated at 60 °C for 3 h. After the reaction, the resulting AgCl precipitate was removed through centrifugation at 8000 rpm for 15 min. The concentration of the synthesized compound was determined using inductively coupled plasma mass spectrometry (ICP-MS) using an Agilent 7500 instrument (Santa Clara, CA, USA).

### 3.4. Preparation of Lipo-Coated CaCO_3_/Cisplatin Hybrid Watermelon-Shaped Nanoparticles (LCa/C@B)

The synthesis of LCa/C is schematically illustrated in Figure 1A. First, two copies of a 4 mL inverse microemulsion were prepared using Triton X-100: Hexanol: Cyclohexane: H_2_O at a ratio of 1.02:1.04:3.94:0.3 (*v*/*v*) and stirred for 5 min until uniform. Subsequently, 200 μL of 200 mM CaCl_2_ or 200 μL of 200 mM cisplatin prodrug and 200 μL of 200 mM Na_2_CO_3_ were added to the respective inverse microemulsion, and DOPA (100 μL, 20 mM) was then added to the microemulsion containing the cisplatin precursor. The mixture was stirred for 20 min. The two types of emulsions were then mixed and reacted for 24 h. After that, 16.0 mL of ethanol was added to the microemulsion, and the mixture was centrifuged at 12,000× *g* for at least 15 min to remove the cyclohexane and surfactants. After being extensively washed with ethanol 2–3 times, the pellets were re-dispersed in 3.0 mL of chloroform to obtain DCa/C nanoparticles. To prepare LCa/C, 28 mg of DOTAP, 21.3 mg of cholesterol, and 12.6 mg of mPEG-DSPE (molar ratio 40:55:5) and 1.0 mL of DCa/C nanoparticle core were dispersed in 5.0 mL of chloroform. After chloroform was removed by rotary evaporation, residual lipids were dispersed in 2.0 mL of ddH_2_O to generate LCa/C. To prepare LCa/C@B, LCa/C and Bmi1 siRNA were mixed at a *w*/*w* (weight LCa/C/weight siRNA) ratio of more than 200:1 in RNase-free H_2_O by adding a stock solution of LCa/C into a Bmi1 siRNA solution. The samples were vortexed for 2–3 min and then incubated at room temperature for 30 min to ensure the formation of LCa/C@B nanocapsules.

### 3.5. Drug Loading Determination

The concentration of cisplatin was determined using Inductively Coupled-Plasma Mass Spectrometry (ICP-MS) after lysing the sample in a Hydrofluoric acid (HF) solution [62].

### 3.6. Nanoparticle Characterization

The particle size and zeta potential of the nanoparticles were measured in their aqueous solution using Zeta PALS (Zeta Potential Analyzer, Brookhaven Instruments Corporation, Austin, TX, USA) following the manufacturer’s instructions. All measurements were conducted at room temperature. Each parameter was measured three times, and the average values and standard deviations were calculated. The shape of the nanoparticles was analyzed using transmission electron microscopy (TEM, JEOL 1010, Tokyo, Japan). For TEM analysis at 100 kV, the powder sample of nanoparticles was dispersed in ethanol and dried on a copper grid. Elemental analysis was performed using techniques such as Energy Dispersive X-ray (EDX).

### 3.7. In Vitro Acid-Responsiveness Release of Cisplatin Under Simulated Conditions

To examine the acid-responsiveness, one milliliter of LCa/C was placed inside a dialysis bag with a molecular weight cut-off of 2000 Da. The dialysis bag was then immersed in 20 mL of PBS containing 0.5% tween 80 at different pH values (pH 6.5, 6.8, and 7.4) and maintained at a temperature of 37 °C. The released platinum (Pt) was quantified using ICP-MS.

### 3.8. Intracellular Uptake of Nanoparticles

To investigate the intracellular uptake of nanoparticles, HepG2 and HepG2/MDR cells were cultured in 24-well plates and treated with different groups (equivalent to 2.5 μg/mL cisplatin) for 48 h. After treatment, the cells were digested with 70% HNO_3_ and diluted in water to achieve a final acid concentration of 2%. The concentration of cisplatin in the solution was determined using ICP-MS. To detect the fluorescence of LCa/C@B (Bmi1 siRNA labeled with FITC), we incubated the nanoparticles for 24 h after adsorption, and then incubated them with tumor cells for 1, 4, and 8 h, respectively. Following that, the medium was replaced with fresh medium, and the fluorescence was observed under a fluorescence microscope.

### 3.9. Cytotoxicity Determination

To assess the cytotoxicity of the drugs, tumor cells were cultured in 96-well plates and treated with different concentrations of the respective drugs. Following a 24 h incubation period, cell survival was measured using a CCK-8 kit (Dojindo Laboratories, Kumamoto, Japan) in accordance with the manufacturer’s instructions. Cell viability was calculated based on the assay results.

### 3.10. Cell Cycle Analyses

For cell cycle analysis by FACS, HepG2 and HepG2/MDR cells were seeded at a density of 4 × 10^5^ cells per well or 70% confluency in DMEM with 10% FBS in 6-well plates and allowed to attach overnight. The culture medium was then replaced with fresh DMEM containing 10% FBS, and the cells were treated with PBS, free cisplatin (5 μM), LC (5 μM cisplatin), LCa/C (5 μM cisplatin), or LCa/C@B (5 μM cisplatin and 50 nM Bmi1 siRNA) for 24 h. After the treatment, the cells were harvested by trypsinization, collected, and fixed in 70% ethanol at 4 °C overnight. Following fixation, the cells were washed and suspended in 200 μL PBS. Subsequently, 5 μL of RNase (20 mg/mL) was added to the cell suspension and incubated at 37 °C for 30 min. The cells were then stained with 20 μL of propidium iodide (500 μg/mL, KeyGen Biotech Co. Ltd., Nanjing, China) at 4 °C for 30 min.

### 3.11. Flow Cytometry

For the analysis of CSC marker FACS detection, HepG2 and HepG2/MDR cells treated with different drugs were incubated with Hoechst 33342 (20 μg/mL) for 1.5 h at 37 °C. To analyze the expression of CD133, phycoerythrin-conjugated CD133/1 (Miltenyi Biotec, Bergisch Gladbach, Germany) was added to the cells. After eliminating the dead cells with propidium iodide, the cells were analyzed using FlowJo 10.8.1 software.

### 3.12. Western Blotting Analysis

Following treatment with different formulations, HepG2/MDR cells were collected and lysed using a cell lysis solution. The resulting lysates were subjected to high-speed centrifugation (12,000 rpm), and the supernatant containing the proteins was collected. Separation of the proteins was performed using a 10% polyacrylamide gel. Subsequently, the separated proteins were transferred onto a PVDF membrane, which was then blocked using 5% skim milk for 1 h to prevent non-specific binding. Monoclonal antibodies against specific proteins were used for immunoblotting. The antibodies used included CD133 (1:1000, Cell Signaling, Danvers, MA, USA), Bmi1 (1:1000, Cell Signaling), and Tubulin (1:1000, Abcam, Cambridge, UK). The membrane was incubated with the primary antibodies overnight at a specified dilution. Following incubation, the membrane was washed three times with TBST (PBS with 0.1% Tween-20) to remove unbound antibodies. Then, the membrane was incubated with the appropriate secondary antibody for 1 h. After another round of washing with TBST, an enhanced chemiluminescence system (Perkin Elmer, Waltham, MA, USA) was used for image development and visualization of the protein bands.

### 3.13. Mice

C57BL/6J mice (4–6 weeks old) were obtained from Beijing Huafukang Bioscience Technology Co., Ltd. (Beijing, China). The C57BL/6J mice were housed in filter-topped cages with standard rodent chow and water available ad libitum, under a 12 h light/dark cycle.

### 3.14. Primary HCC Mouse Model

To establish the primary HCC mouse model, a double overexpression gene of Akt/Ras was generated to induce primary HCC, and the anti-tumor effect of different drugs was evaluated. All gene constructs of hyperactive transposase (pCMV/SB), AKT (myr-AKT/pT3EF1α), and Ras (pCaggs-RasV12) were provided by Professor Xin Chen (University of California at San Francisco). The GenElute Endotoxin-free Plasmid Maxiprep Kit (Sigma, Saint Louis, MO, USA) was used to purify the plasmids before injecting them into the mice.

The high-pressure injection technique via the tail vein was performed following the protocol described in previous reports. Briefly, C57BL/6J mice were injected with 2 mL of saline containing NRasV12/pT2-CAGGS (5 μg), myr-AKT/pT3EF1α (5 μg), and pCMV/SB (0.4 μg) within 5–10 s via the tail vein. After approximately six weeks, HCC tumors were formed in the liver due to the up-regulation of AKT/Ras gene expression, and confirmation was obtained by bimanual palpation and dissection [62,63]. Animals were randomly assigned into five treatment groups. Investigators performing tumor size measurement, bioluminescence imaging, histology scoring, and data analysis were blinded to group allocation. Humane endpoints included over 20% weight loss, severe tumor ulceration, or uncontrollable tumor growth. At the end of the experiment, the mice were euthanized with 5% isoflurane anesthesia and cervical dislocation.

Mice were anesthetized by isoflurane gas (2%, inhalation) and given an i.p. injection of D-firefly luciferin (240 mg/kg) (Gold Biotechnology, St. Louis, MO, USA). Mice were placed into the chamber of a Xenogen IVIS 200 imaging system and bioluminescence images were obtained under isoflurane anesthesia using 1 min exposures beginning 10 min after D-luciferin injection. The images were quantitatively analyzed by Living Image version 4.3.1 image analysis software (Caliper Life Sciences, Hopkinton, MA, USA). Total bioluminescence measurements (photon/s) were quantified over a contour drawn around liver tumor regions. Results were repeated two to three times in independent animals. All in vivo images are scaled to maximum intensity of 1 × 10^5^ photons/s/cm^2^/sr.

### 3.15. Statistical Analysis

Student’s *t*-test (SPSS 22.0 Software, Chicago, IL, USA) was used for comparisons between two groups. One-way ANOVA (GraphPad Prism 5.0, San Diego, CA, USA) with Dunnett’s post-test was used to compare multiple groups.

## 4. Conclusions

In this study, we synthesized acid-responsive lipid-coated CaCO_3_/Cisplatin “Watermelon-shaped” nanoparticles (LCa/C) and utilized them in combination with Bmi1 siRNA (LCa/C@B) to address cisplatin-resistance in HCC. The efficacy of LCa/C@B was assessed both in vitro and in vivo. The results demonstrated that LCa/C@B effectively improved acquired and intrinsic cisplatin resistance and enhanced cisplatin sensitivity in resistant HCC cells by comprehensively inhibiting HCC CSCs. Furthermore, LCa/C@B inhibited gene-driven HCC in situ, leading to improved chemotherapy outcomes. These findings suggest that the simultaneous regulation of acquired and intrinsic HCC resistance mechanisms through novel drug delivery systems, combined with tumor stem cell inhibitors, holds promise as a therapeutic approach to overcome HCC resistance.

## Data Availability

The original contributions presented in this study are included in the article/Supplementary Material. Further inquiries can be directed to the corresponding authors.

## Supplementary Materials

The following supporting information can be downloaded at: https://www.mdpi.com/article/10.3390/ph19010022/s1, Table S1: Polydispersity index (PDI) of DC, DCa/C, LCa/C, and LCa/C@B nanoparticles measured by DLS; Figure S1: Western blotting of Bmi1 protein expression in HepG2, HepG2/MDR, and free cisplatin, LC, LCa/C, and LCa/C@B-treated HepG2/MDR cells.

## References

[1] Yang J.D., Roberts L.R. (2010). Hepatocellular carcinoma: A global view. Nat. Rev. Gastroenterol. Hepatol..

[2] Yang J.D., Hainaut P., Gores G.J., Amadou A., Plymoth A., Roberts L.R. (2019). A global view of hepatocellular carcinoma: Trends, risk, prevention and management. Nat. Rev. Gastroenterol. Hepatol..

[3] Zhang C., Chen Y., Han J., Liu R., Liu C., Zhao Y., Liu Y. (2025). Ultrasound nanobubble-based combinational strategies of loaded miR-107-3p and CD133 Ab for anti-PD-L1 and anti-hepatocellular cancer stem cells. Int. J. Pharm..

[4] Liu M., Jiang L., Guan X.Y. (2014). The genetic and epigenetic alterations in human hepatocellular carcinoma: A recent update. Protein Cell.

[5] Unagolla J.M., Das S., Flanagan R., Oehler M., Menon J.U. (2024). Targeting chronic liver diseases: Molecular markers, drug delivery strategies and future perspectives. Int. J. Pharm..

[6] Lin X., Ojo D., Wei F., Wong N., Gu Y., Tang D. (2015). A Novel Aspect of Tumorigenesis-BMI1 Functions in Regulating DNA Damage Response. Biomolecules.

[7] Mekala J.R., Vaishnave S. (2018). BMI1 and PTEN are key determinants of breast cancer therapy: A plausible therapeutic target in breast cancer. Gene.

[8] Jia L., Zhang W., Wang C.Y. (2020). BMI1 Inhibition Eliminates Residual Cancer Stem Cells after PD1 Blockade and Activates Antitumor Immunity to Prevent Metastasis and Relapse. Cell Stem Cell.

[9] Li Y., Tian Z., Tan Y., Lian G., Chen S., Chen S., Li J., Li X., Huang K., Chen Y. (2020). Bmi-1-induced miR-27a and miR-155 promote tumor metastasis and chemoresistance by targeting RKIP in gastric cancer. Mol. Cancer.

[10] Zhang L., Qiang J., Yang X., Wang D., Rehman A.U., He X., Chen W., Sheng D., Zhou L., Jiang Y.Z. (2020). IL1R2 Blockade Suppresses Breast Tumorigenesis and Progression by Impairing USP15-Dependent BMI1 Stability. Adv. Sci..

[11] Zhou T., Wang L., Zhu K.Y., Dong M., Xu P.F., Chen Y., Chen S.J., Chen Z., Deng M., Liu T.X. (2011). Dominant-negative C/ebpalpha and polycomb group protein Bmi1 extend short-lived hematopoietic stem/progenitor cell life span and induce lethal dyserythropoiesis. Blood.

[12] Molofsky A.V., Pardal R., Iwashita T., Park I.K., Clarke M.F., Morrison S.J. (2003). Bmi-1 dependence distinguishes neural stem cell self-renewal from progenitor proliferation. Nature.

[13] Tian H., Biehs B., Warming S., Leong K.G., Rangell L., Klein O.D., de Sauvage F.J. (2011). A reserve stem cell population in small intestine renders Lgr5-positive cells dispensable. Nature.

[14] Dirks P. (2007). Bmi1 and cell of origin determinants of brain tumor phenotype. Cancer Cell.

[15] Sherr C.J. (2001). The INK4a/ARF network in tumour suppression. Nat. Rev. Mol. Cell Biol..

[16] Zhu S., Zhao D., Yan L., Jiang W., Kim J.S., Gu B., Liu Q., Wang R., Xia B., Zhao J.C. (2018). BMI1 regulates androgen receptor in prostate cancer independently of the polycomb repressive complex 1. Nat. Commun..

[17] Bhattacharya R., Mustafi S.B., Street M., Dey A., Dwivedi S.K. (2015). Bmi-1: At the crossroads of physiological and pathological biology. Genes. Dis..

[18] Frank N.Y., Schatton T., Frank M.H. (2010). The therapeutic promise of the cancer stem cell concept. J. Clin. Investig..

[19] Rahimkhoei V., Akbari A., Jassim A.Y., Hussein U.A., Salavati-Niasari M. (2025). Recent advances in targeting cancer stem cells by using nanomaterials. Int. J. Pharm..

[20] Zhou J.N., Zhang B., Wang H.Y., Wang D.X., Zhang M.M., Zhang M., Wang X.K., Fan S.Y., Xu Y.C., Zeng Q. (2022). A Functional Screening Identifies a New Organic Selenium Compound Targeting Cancer Stem Cells: Role of c-Myc Transcription Activity Inhibition in Liver Cancer. Adv. Sci..

[21] He C., Jaffar Ali D., Qi Y., Li Y., Sun B., Liu R., Sun B., Xiao Z. (2023). Engineered extracellular vesicles mediated CRISPR-induced deficiency of IQGAP1/FOXM1 reverses sorafenib resistance in HCC by suppressing cancer stem cells. J. Nanobiotechnol..

[22] Sagar J., Chaib B., Sales K., Winslet M., Seifalian A. (2007). Role of stem cells in cancer therapy and cancer stem cells: A review. Cancer Cell Int..

[23] Persi E., Duran-Frigola M., Damaghi M., Roush W.R., Aloy P., Cleveland J.L., Gillies R.J., Ruppin E. (2018). Systems analysis of intracellular pH vulnerabilities for cancer therapy. Nat. Commun..

[24] Cairns R.A., Harris I.S., Mak T.W. (2011). Regulation of cancer cell metabolism. Nat. Rev. Cancer.

[25] Begicevic R.R., Falasca M. (2017). ABC Transporters in Cancer Stem Cells: Beyond Chemoresistance. Int. J. Mol. Sci..

[26] Shibue T., Weinberg R.A. (2017). EMT, CSCs, and drug resistance: The mechanistic link and clinical implications. Nat. Rev. Clin. Oncol..

[27] Wang Y., Gong X., Li J., Wang H., Xu X., Wu Y., Wang J., Wang S., Li Y., Zhang Z. (2021). M2 macrophage microvesicle-inspired nanovehicles improve accessibility to cancer cells and cancer stem cells in tumors. J. Nanobiotechnol..

[28] Lee T.K., Guan X.Y., Ma S. (2022). Cancer stem cells in hepatocellular carcinoma—From origin to clinical implications. Nat. Rev. Gastroenterol. Hepatol..

[29] Holze C., Michaudel C., Mackowiak C., Haas D.A., Benda C., Hubel P., Pennemann F.L., Schnepf D., Wettmarshausen J., Braun M. (2018). Oxeiptosis, a ROS-induced caspase-independent apoptosis-like cell-death pathway. Nat. Immunol..

[30] Han J., Won M., Kim J.H., Jung E., Min K., Jangili P., Kim J.S. (2020). Cancer stem cell-targeted bio-imaging and chemotherapeutic perspective. Chem. Soc. Rev..

[31] Zarubin T., Han J. (2005). Activation and signaling of the p38 MAP kinase pathway. Cell Res..

[32] Romagosa C., Simonetti S., Lopez-Vicente L., Mazo A., Lleonart M.E., Castellvi J., Ramon y Cajal S. (2011). p16(Ink4a) overexpression in cancer: A tumor suppressor gene associated with senescence and high-grade tumors. Oncogene.

[33] Cheung E.C., Vousden K.H. (2022). The role of ROS in tumour development and progression. Nat. Rev. Cancer.

[34] Wang H., Gao Z., Liu X., Agarwal P., Zhao S., Conroy D.W., Ji G., Yu J., Jaroniec C.P., Liu Z. (2018). Targeted production of reactive oxygen species in mitochondria to overcome cancer drug resistance. Nat. Commun..

[35] Ding S., Li C., Cheng N., Cui X., Xu X., Zhou G. (2015). Redox Regulation in Cancer Stem Cells. Oxid. Med. Cell Longev..

[36] Zhang C., Wang X., Du J., Gu Z., Zhao Y. (2021). Reactive Oxygen Species-Regulating Strategies Based on Nanomaterials for Disease Treatment. Adv. Sci..

[37] Courtnay R., Ngo D.C., Malik N., Ververis K., Tortorella S.M., Karagiannis T.C. (2015). Cancer metabolism and the Warburg effect: The role of HIF-1 and PI3K. Mol. Biol. Rep..

[38] Kang H., Kim B., Park J., Youn H., Youn B. (2023). The Warburg effect on radioresistance: Survival beyond growth. Biochim. Biophys. Acta Rev. Cancer.

[39] Shamsi M., Saghafian M., Dejam M., Sanati-Nezhad A. (2018). Mathematical Modeling of the Function of Warburg Effect in Tumor Microenvironment. Sci. Rep..

[40] Gu J., Zhao G., Yu J., Xu P., Yan J., Jin Z., Chen S., Wang Y., Zhang L.W., Wang Y. (2022). Injectable pH-responsive hydrogel for combinatorial chemoimmunotherapy tailored to the tumor microenvironment. J. Nanobiotechnol..

[41] Gao Y., Zhao Q., Xiao M., Huang X., Wu X. (2021). A versatile photothermal vaccine based on acid-responsive glyco-nanoplatform for synergistic therapy of cancer. Biomaterials.

[42] Liu Y., Si L., Jiang Y., Jiang S., Zhang X., Li S., Chen J., Hu J. (2025). Design of pH-Responsive Nanomaterials Based on the Tumor Microenvironment. Int. J. Nanomed..

[43] Larroque-Lombard A.L., Chatelut E., Delord J.P., Imbs D.C., Rochaix P., Jean-Claude B., Allal B. (2021). Design and Mechanism of Action of a New Prototype of Combi-Molecule “Programed” to Release Bioactive Species at a pH Range Akin to That of the Tumor Microenvironment. Pharmaceuticals.

[44] Ma Y., Lai P., Sha Z., Li B., Wu J., Zhou X., He C., Ma X. (2025). TME-responsive nanocomposite hydrogel with targeted capacity for enhanced synergistic chemoimmunotherapy of MYC-amplified osteosarcoma. Bioact. Mater..

[45] Li J., Dai Y., Wang T., Zhang X., Du P., Dong Y., Jiao Z. (2025). Polyphenol-based pH-responsive nanoparticles enhance chemo-immunotherapy in pancreatic cancer. J. Control. Release.

[46] Zhang S., Zhu P., He J., Dong S., Li P., Zhang C.Y., Ma T. (2020). TME-Responsive Polyprodrug Micelles for Multistage Delivery of Doxorubicin with Improved Cancer Therapeutic Efficacy in Rodents. Adv. Healthc. Mater..

[47] Su Q., Wang Z., Li P., Wei X., Xiao J., Duan X. (2024). pH and ROS Dual-Responsive Autocatalytic Release System Potentiates Immunotherapy of Colorectal Cancer. Adv. Healthc. Mater..

[48] Yin C., Wei Z.J., Long K., Sun M., Zhang Z., Wang Y., Wang W., Yuan Z. (2024). pH-Responsive Persistent Luminescent Nanosystem with Sensitized NIR Imaging and Ratiometric Imaging Modes for Tumor Surgery Navigation. ACS Appl. Mater. Interfaces.

[49] Zhang Y.F., Chen K., Zhu Y.Q., Liu L., Kong X.T., Dai Y., Fu X.J., Li Y.L., Liu M.H., Zhang D. (2025). Preparation of cancer cell membrane-coated Gambogic acid-loaded pH-sensitive liposomes to enhance targeted anti-hepatocellular carcinoma effect. Drug Deliv. Transl. Res..

[50] Zhou H., Yang J., Li Z., Feng J., Duan X., Yan C., Wen G., Qiu X., Shen Z. (2024). Hollow mesoporous calcium peroxide nanoparticles for drug-free tumor calcicoptosis therapy. Acta Biomater..

[51] Yang X., Zhang H., Wu Z., Chen Q., Zheng W., Shen Q., Wei Q., Shen J.W., Guo Y. (2024). Tumor therapy utilizing dual-responsive nanoparticles: A multifaceted approach integrating calcium-overload and PTT/CDT/chemotherapy. J. Control. Release.

[52] Li W., Wang H., Liu Y., Li B., Wang F., Ye P., Xu Y., Lai Y., Yang T. (2024). “Trinity” Comprehensively Regulates the Tumor Microenvironment of Lipid-Coated CaCO(3)@CuO(2) Nanoparticles Induces “Cuproptosis” in HCC. ACS Appl. Mater. Interfaces.

[53] Guo Z., Xing R., Zhao M., Li Y., Lu H., Liu Z. (2021). Controllable Engineering and Functionalizing of Nanoparticles for Targeting Specific Proteins towards Biomedical Applications. Adv. Sci..

[54] Guo S., Wang Y., Miao L., Xu Z., Lin C.M., Zhang Y., Huang L. (2013). Lipid-coated Cisplatin nanoparticles induce neighboring effect and exhibit enhanced anticancer efficacy. ACS Nano.

[55] Chiba T., Seki A., Aoki R., Ichikawa H., Negishi M., Miyagi S., Oguro H., Saraya A., Kamiya A., Nakauchi H. (2010). Bmi1 promotes hepatic stem cell expansion and tumorigenicity in both Ink4a/Arf-dependent and -independent manners in mice. Hepatology.

[56] Biehs B., Hu J.K., Strauli N.B., Sangiorgi E., Jung H., Heber R.P., Ho S., Goodwin A.F., Dasen J.S., Capecchi M.R. (2013). BMI1 represses Ink4a/Arf and Hox genes to regulate stem cells in the rodent incisor. Nat. Cell Biol..

[57] Liu J., Cao L., Chen J., Song S., Lee I.H., Quijano C., Liu H., Keyvanfar K., Chen H., Cao L.Y. (2009). Bmi1 regulates mitochondrial function and the DNA damage response pathway. Nature.

[58] Craig A.J., von Felden J., Garcia-Lezana T., Sarcognato S., Villanueva A. (2020). Tumour evolution in hepatocellular carcinoma. Nat. Rev. Gastroenterol. Hepatol..

[59] Xia H., Li F., Hu X., Park W., Wang S., Jang Y., Du Y., Baik S., Cho S., Kang T. (2016). pH-Sensitive Pt Nanocluster Assembly Overcomes Cisplatin Resistance and Heterogeneous Stemness of Hepatocellular Carcinoma. ACS Cent. Sci..

[60] Shen D.W., Pouliot L.M., Hall M.D., Gottesman M.M. (2012). Cisplatin resistance: A cellular self-defense mechanism resulting from multiple epigenetic and genetic changes. Pharmacol. Rev..

[61] Zhou L., Yu K.H., Wong T.L., Zhang Z., Chan C.H., Loong J.H., Che N., Yu H.J., Tan K.V., Tong M. (2022). Lineage tracing and single-cell analysis reveal proliferative Prom1+ tumour-propagating cells and their dynamic cellular transition during liver cancer progression. Gut.

[62] Li W., Solenne T., Wang H., Li B., Liu Y., Wang F., Yang T. (2023). Core-shell cisplatin/SiO_2_ nanocapsules combined with PTC-209 overcome chemotherapy-Acquired and intrinsic resistance in hepatocellular carcinoma. Acta Biomater..

[63] Zheng H.C., Xue H., Yun W.J. (2023). An overview of mouse models of hepatocellular carcinoma. Infect. Agent. Cancer.

