# Supplementary material for: Dual-Targeting CSC Therapy: Acid-Responsive Cisplatin/CaCO3@siRNA Nanoplatform Overcomes HCC Chemoresistance
Source: Pharmaceuticals (Basel). 2025 Dec 22;19(1):22. doi: 10.3390/ph19010022 (PMC12845244; doi:10.3390/ph19010022)
Supplement: Supplementary file 1 [file pharmaceuticals-19-00022-s001.zip › pharmaceuticals-4029764-supplementary.pdf]

Supporting information

Dual-Targeting CSC Therapy: Acid-Responsive Cisplatin/CaCO3@siRNA Nanoplatform Overcomes HCC Chemoresistance

Fei Wang [2,†], Ming Lin [4,†], Yong Liu [3], Han Wang [3], Bin Li [3], Tan Yang [3,*] and Weijie Li [1,*]

1 Department of Pharmacy, Tongji Hospital, Tongji Medical College, Huazhong University of Science and Technology, Wuhan 430030, China
2 Department of Oncology, Renmin Hospital of Wuhan University, Wuhan 430060, China; feiwang2021@whu.edu.cn
3 School of Pharmacy, Tongji Medical College, Huazhong University of Science and Technology, Wuhan 430030, China; liuyongswe@163.com (Y.L.); 15971506312@163.com (H.W.); libin@youdubio.com (B.L.)
4 Department of Pediatrics, Union Hospital, Tongji Medical College, Huazhong University of Science and Technology, Wuhan 430022, China; linming@hust.edu.cn
* Correspondence: yangtan0120@hust.edu.cn (T.Y.); 13308647450@163.com (W.L.)
† These authors contributed equally to this work.

	PDI
**DC**	0.188 ± 0.031
**DCa/C**	0.215 ± 0.027
**LCa/C**	0.218 ± 0.017
**LCa/C@B**	0.222 ± 0.031

Table S1. Polydispersity index (PDI) of DC, DCa/C, LCa/C, and LCa/C@B nanoparticles measured by DLS.

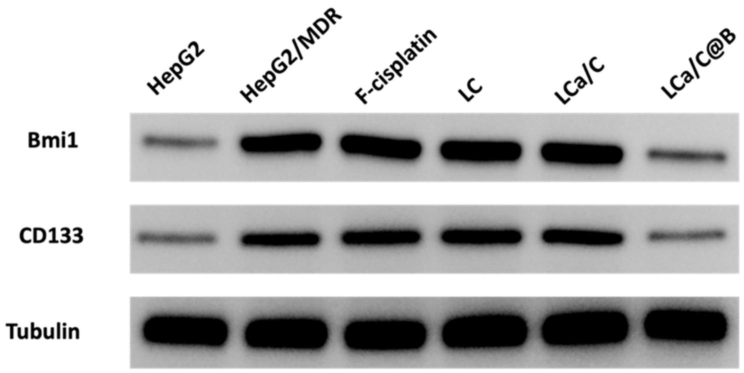

Figure S1. Western blotting of Bmi1 protein expression in HepG2, HepG2/MDR, and free cisplatin, LC, LCa/C, and LCa/C@B-treated HepG2/MDR cells.
